# Therapeutic Targeting of Autophagy for Renal Cell Carcinoma Therapy

**DOI:** 10.3390/cancers12051185

**Published:** 2020-05-07

**Authors:** Trace M. Jones, Jennifer S. Carew, Steffan T. Nawrocki

**Affiliations:** Division of Translational and Regenerative Medicine, Department of Medicine and The University of Arizona Cancer Center, Tucson, AZ 85724, USA; tracejones@email.arizona.edu (T.M.J.); jcarew@email.arizona.edu (J.S.C.)

**Keywords:** renal cell carcinoma, autophagy, hydroxychloroquine, chloroquine, ROC-325

## Abstract

Kidney cancer is the 7th most prevalent form of cancer in the United States with the vast majority of cases being classified as renal cell carcinoma (RCC). Multiple targeted therapies have been developed to treat RCC, but efficacy and resistance remain a challenge. In recent years, the modulation of autophagy has been shown to augment the cytotoxicity of approved RCC therapeutics and overcome drug resistance. Inhibition of autophagy blocks a key nutrient recycling process that cancer cells utilize for cell survival following periods of stress including chemotherapeutic treatment. Classic autophagy inhibitors such as chloroquine and hydroxychloroquine have been introduced into phase I/II clinical trials, while more experimental compounds are moving forward in preclinical development. Here we examine the current state and future directions of targeting autophagy to improve the efficacy of RCC therapeutics.

## 1. Introduction

It is estimated that over 73,000 new cases of renal cancer will be diagnosed in the United States this year, with upwards of 14,000 individuals succumbing to their disease [1]. The most common malignancy of the kidney is renal cell carcinoma (RCC), which accounts for 85% of cases [2]. RCC can be divided into three distinct histological subtypes. Clear cell RCC (ccRCC) is the predominant subtype (~75%), with papillary RCC (PRCC) and chromophobe RCC (ChRCC) accounting for ~20% and ~5% of cases, respectively [3]. Disease stage at the time of diagnosis is the most important factor when considering the best course of treatment. Localized disease, generally TNM stage I or II, has a positive prognosis with a 5-year relative survival rate of 92.6% [4]. Localized neoplasms can be effectively treated with either a partial or radical nephrectomy, depending on the location of the primary mass [5]. After successful surgery, patients are often simply surveyed for signs of recurrence. It is estimated that 20–30% of patients who have undergone a successful nephrectomy will experience a recurrence, often presenting between one to three years following surgery [6]. Following a relapse, patients often undergo treatment with chemotherapy or immunotherapy depending on their histologic subtype. Patients presenting with regionally or distantly invasive tumors have a less favorable prognosis. A nephrectomy is still the primary first line treatment. However, patients are often administered chemotherapy, immunotherapy, or enrolled in a clinical trial in order to manage metastases and tumors that are surgically unresectable [5].

**Figure 1:**
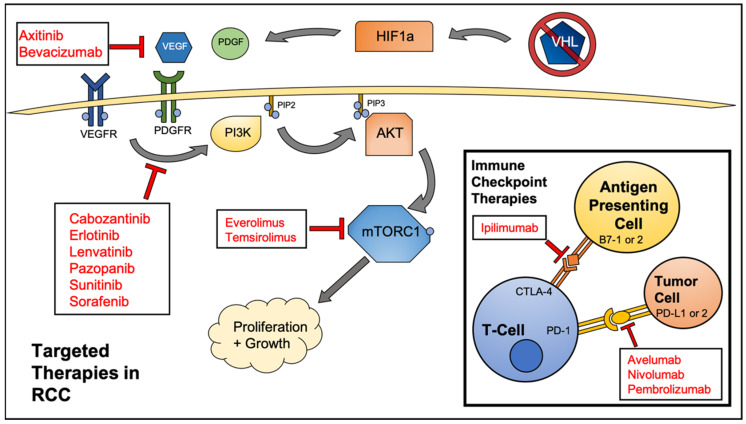
Federal Drug Administration (FDA) approved agents to treat renal cell carcinoma (RCC). Various kinase and mammalian target of rapamycin (mTOR) inhibitors are amongst the most common drugs used, however, immune checkpoint inhibitors are becoming a mainstay of RCC treatment.

Given that metastatic, relapsed, and surgically unresectable tumors must be treated by systemic chemotherapy or immunotherapy, great interest has been shown in the past decade in developing targeted therapeutics for RCC. The most commonly mutated gene in RCC is the *von Hippel-Lindau (VHL)* tumor suppressor gene. Approximately 50% of RCC cases contain a mutation in this gene, with an additional 20% of cases presenting with a hypermethylated gene [7]. The VHL protein is an E3 ubiquitin ligase that controls the conjugation of ubiquitin molecules onto hypoxia-inducible factors (HIFs), proteins that are vital to the cellular hypoxia response pathway. Upon ubiquitylation, HIFs are processed and degraded through the ubiquitin proteasome pathway. Without a functional copy of *VHL*, HIFs are free to translocate to the nucleus and activate transcription of HIF responsive genes. A few of these HIF responsive genes code for vascular endothelial growth factor (VEGF), platelet-derived growth factor B (PDGF-B), transforming growth factor alpha (TGFα), and glucose transporter 1 (GLUT1) [7]. The overexpression of these factors is often a driving force in RCC tumorigenesis. In addition to *VHL*, genes involved in the mammalian target of rapamycin (mTOR) pathway are mutated in 28% of RCC cases [8,9]. These include genes encoding for phosphatidylinositol-3-kinase (PI3K), phosphatase and tensin homolog (PTEN), protein kinase B (AKT), and mTOR itself. These frequent mutation profiles provide the rationale for therapeutically targeting various receptor tyrosine kinases (RTKs) and downstream effector proteins currently being developed and used in the clinic (Figure 1).

Although targeted tyrosine kinase and mTOR inhibitors are effective first-line treatment options, many, if not all, cases of RCC will eventually become resistant to these drugs. The median time to a resistant tumor phenotype is 6–15 months depending on the therapeutic regimen [10]. A better understanding of the mechanistic drivers of drug resistance in RCC will facilitate the development of new and more effective treatment options for the relapsed/refractory patient population.

A hallmark of cancer is evasion of the immune response [11]. Cancer cells are capable of evading immune surveillance by expressing various signals that act as “off” switches to T-cells and natural killer (NK) cells. The most well-characterized of these signals are cytotoxic T-lymphocyte associated protein 4 (CTLA-4) and programmed cell death ligand 1 (PD-L1). When these surface proteins come in contact with the appropriate receptor on T-cells, they effectively trick the lymphocyte into recognizing the cancer cell as normal self-cells. Given this, an immense amount of energy has been dedicated to developing monoclonal antibody therapies to block the binding of cancer cell expressed PD-L1 and CTLA-4 allowing the immune cells to recognize the tumor cells as a foreign entity. These immune checkpoint inhibitor therapies enable the immune system to both eliminate tumor cells and also develop a lasting immune response. A persistent remission state is observed in two thirds of patients who experience an initial response to these therapies [12]. Importantly, immune checkpoint inhibitors have demonstrated significant efficacy in patients with RCC.

## 2. Targeted Therapeutics for RCC

For multiple decades, the standard therapy for RCC patients was a regimen of cytokines. While more effective than traditional chemotherapy options, interferon-alpha, and interleukin-2 as single agents or in combination yielded low response rates in patients with the combination generating an 18.6% response rate [13]. In addition, cytokine therapy was often associated with severe adverse effects and the incidence of comorbidities was high. With the advent of targeted therapies for cancer patients came an influx of approved therapeutics for RCC patients. There are now a multitude of Federal Drug Administration (FDA)-approved targeted treatments for RCC. The target-specific therapies can roughly be broken down into three distinct categories: small molecule kinase inhibitors, mTOR inhibitors, and monoclonal antibodies. The monoclonal antibodies frequently used to treat advanced RCC can be further classified as immune checkpoint inhibitors and non-immunomodulatory antibodies. A listing of targeted therapies approved for use in RCC can be found in Table 1.

Due to the frequent inactivation of VHL and subsequent overexpression of HIF1a, RCC often presents as a highly vascularized tumor type. Hence, many of the small molecule therapeutics approved for use in RCC target various effectors in the angiogenesis pathway (VEGF, VEGFR). The goal of these drugs is to abrogate the formation of new blood vessels in the tumor microenvironment via growth factor withdrawal, which deprives the cancer cells of oxygen and nutrients that are needed to fuel their growth and survival. Many of these therapies provide an initial response. However, oxygen deprivation and nutrient withdrawal activates various stress response pathways in the cancer cell. Autophagy is one such stress response, and allows for survival during periods of therapeutic insult.

## 3. Autophagy

### 3.1. Molecular Mechanisms of Autophagy

Autophagy is a catabolic process by which cells internally break down and recycle cellular components through non-specific, lysosome-mediated degradation. Autophagy is highly conserved in eukaryotes and is a vital mechanism to mediate cellular stress and damage that results from hypoxia and starvation, as well as therapeutic intervention. Mammalian target of rapamycin complex 1 (mTORC1) is often regarded as the master regulatory kinase of cellular metabolism. Activation of mTORC1 is generally thought of as a pro-proliferation signal. mTORC1 activity can be stimulated from activated upstream tyrosine kinases such as PDGFR and VEGFR, often through the phosphoinositide 3 kinase (PI3K)/ protein kinase B (AKT) pathway. This mechanism of mTORC1 activation is especially important in RCC, given the genes encoding the proteins involved are frequently mutated. Importantly, activated mTORC1 is responsible for adding an inhibitory phosphate at Serine 757 to the Unc-51-like kinase (ULK1) complex, which prevents the initiation of autophagy [30]. Both direct and indirect inhibition of mTORC1 leads to potent activation of the autophagy inducer ULK1, which in turn promotes activity of the Beclin1-Vacuolar protein sorting 34 (VPS34) complex. The Beclin1-VPS34 complex is vital to the nucleation of the premature phagophore.

Maturation of the phagophore into a complete autophagosome involves elongation of the vesicle’s lipid membrane. This process is regulated by a complex containing autophagy-related 12, autophagy-related 5 and autophagy-related 16L (ATG12-ATG5-ATG16L). Another crucial protein involved in the elongation of the autophagosome membrane is microtubule-associated protein 1A/1B light chain 3 (LC3). Cytosolic LC3 is referred to as LC3-I. LC3-I is conjugated in a ubiquitin-like fashion to phosphatidylethanolamines (PE) on the autophagosome membrane by the autophagy protein, autophagy-related 7 (ATG7). It must be mentioned that autophagy related 4B (ATG4B), a widely conserved cysteine protease, must first make a specific cleavage to allow conjugation of LC3 [31]. PE-associated LC3 protein is referred to as LC3-II, and is often considered a reliable marker of autophagosome formation and autophagy. LC3-II is vital for cargo recruitment as it binds Sequestosome-1 (p62) to the autophagosome membrane. p62 is responsible for binding misfolded proteins or dysfunctional organelles and subsequently delivering them to the autophagosome for degradation [32]. Finally, the mature autophagosome, with its contents localized and completely enclosed will fuse with the lysosome. The fusion of membranes will release the cargo of the autophagosome into the lysosome where the acidic pH, as well as various enzymes will facilitate their degradation. After degradation has occurred, the remaining molecules are released back into the cytoplasm where they can be used as building blocks for new proteins, organelles, or energy sources. We will specifically focus on the role of autophagy in RCC pathogenesis and its involvement related to emerging targeted therapies in RCC. For a more extensive review of the molecular machinery and regulation of autophagy, refer to the following articles [33,34,35].

### 3.2. Targeting Autophagy to Improve RCC Therapeutic Outcome

Autophagy is an essential lysosomal degradation process that can be used by cancer cells to generate alternative sources of energy via nutrient recycling under stress conditions [36,37]. Although many studies have demonstrated that autophagy may function as a mechanism of tumor suppression through the degradation of defective pre-malignant cells, significant data indicates a key role for autophagic degradation in the maintenance of energy balance under stress conditions including nutrient deprivation and hypoxia [38]. Futhermore, autophagy has emerged as an important mechanism of resistance to radiation, conventional chemotherapy, and targeted anticancer agents due to its ability to enhance the stress tolerance of malignant cells [39,40,41,42,43]. Collectively, these data support a role for autophagy as a promoter of drug resistance and cancer progression as well as a target for therapeutic inhibition. Importantly, several new studies demonstrate that alterations in the autophagy pathway may be particularly relevant for patients with RCC and impact overall survival [44,45].

RCC cell lines inherently exhibit an elevated basal level of autophagy. One study found that across many RCC cell lines, 30–60% of growing cells display prominent LC3-II puncta [46]. This compares to just 1–5% of cells in normal primary kidney cell cultures. Autophagy has been shown to counteract growth factor and nutrient withdrawal and maintain cell viability under stress conditions [47]. Importantly, inhibiting autophagy in RCC increases the efficacy of many therapeutic strategies. Sorafenib, a general RTK inhibitor, shows a significant increase in activity when combined with autophagy inhibitors [48]. The efficacy of AKT/mTOR inhibition is also significantly augmented through the use of a variety of autophagy inhibitors [49]. It has been demonstrated that RCC cells that have adopted an aggressive metastatic phenotype also rely on an increase in cellular autophagic flux. These highly aggressive cells can be rendered sensitive to the mTOR inhibitor temsirolimus by chloroquine, an anti-malarial drug that inhibits autophagy [50]. Successful enhancement of therapeutic efficacy via in vitro autophagy inhibition has provided a solid foundation for the development and clinical testing of autophagy inhibitors in RCC.

## 4. Inhibition of Autophagy for Therapy of RCC

### 4.1. Chloroquine and Hydroxychloroquine

Chloroquine (CQ) and hydroxychloroquine (HCQ) are quinone-containing compounds that have been used to combat malaria for decades. These compounds work via their accumulation in and subsequent deacidification of lysosomes [51]. This deacidification disrupts autophagy as the low pH of lysosomes is a necessity in degrading the cargo of the autophagosome. CQ and HCQ have been repurposed to pharmacologically target autophagy in a broad range of cancer types for over a decade [52]. To date, CQ and HCQ are the only autophagy inhibitors to be evaluated in clinical trials. A number of clinical trials involving the use of CQ or HCQ alone or in combination with standard of care agents for the treatment of many different malignancies are ongoing and completed. However, limited clinical studies have evaluated HCQ in patients with RCC (Table 2).

A recent study in RCC combined the mTOR inhibitor, everolimus, with twice daily doses of HCQ in a metastatic patient population refractory to at least one prior treatment [53]. No dose-limiting toxicities (DLTs) were attributed to the HCQ in the phase I portion of the trial. The median progression-free survival (PFS) for the patient population was 6.3 months, an improvement over the median PFS of 4 months, which was observed in the clinical testing of everolimus alone [21]. A separate phase I trial sought to elucidate the toxicity and efficacy of combining HCQ with an AKT inhibitor, MK-2206, in a multitude of advanced solid tumors [55]. Patients were administered 135–200 mg of MK-2206 weekly in combination with 200–600 mg HCQ twice a day. 31 of the 35 patients enrolled were taken off treatment due to relapsed or progressive disease. In addition, 94% of participants experienced an adverse event (AE) attributed to treatment with MK-2206, while only 13% experienced an AE from the HCQ. Due to high toxicity and the low enrollment on the study, no anti-tumor activity data could be interpreted. One phase I trial has provided an exciting preliminary example of the power of HCQ in combination with vorinostat, an FDA approved histone deacetylase (HDAC) inhibitor [54]. This trial included patients with a variety of advanced solid tumors who had failed conventional therapy. Of these patients, a single person presented with advanced RCC. This particular patient had failed seven previous lines of therapy. A durable, partial response was obtained with a regimen of 400 mg vorinostat and 400 mg HCQ, administered daily. This response was maintained for more than 50 three-week cycles of the drug combination. This remarkable result in an RCC patient has sparked follow-up studies to evaluate tumor characteristics that may be indicative of a positive response to concurrent HDAC and autophagy inhibition.

### 4.2. Lucanthone

In addition to HCQ and CQ, several other agents used for non-cancer indications that disrupt lysosomal function have been repurposed as autophagy inhibitors for cancer therapy [41]. Lucanthone has been used as an anti-schistosome agent for many years and is also being developed as an anticancer agent due to its inhibitory effects on topoisomerase II and AP endonuclease (APE1). In cell culture experiments, lucanthone demonstrated lysosomal disruption and inhibition of autophagy [56]. In addition, strong pro-apoptotic effects were evident in various breast cancer cell lines and the lysosomal protease, cathepsin D, was shown to be an important mediator for the apoptotic effects of lucanthone. The chemical structure of lucanthone has provided clues to the construction of novel, lysosome-targeting agents.

### 4.3. ROC-325

While the clinical benefits of adjuvant CQ and HCQ treatment have not been fully elucidated, a substantial amount of funding and effort has been put forth to develop more efficient autophagy inhibitors. Initial results stemming from the use of CQ or HCQ have indicated that while these drugs partially block the degradation of cellular components in the lysosome, the compounds may not be potent enough to completely shut off the autophagy degradation. Thus, it is essential to generate new and more potent autophagy inhibitors that may improve clinical efficacy. A complete listing of next generation autophagy inhibitors discussed in this review, as well as the cancer types they have been explored in, can be found in Table 3.

ROC-325 is a water-soluble, small molecule developed by our group that shows significantly higher efficacy than HCQ [57,58,59,60]. ROC-325 is a dimeric compound that was designed to contain core motifs of HCQ as well as lucanthone. Much like HCQ, ROC-325 targets the late stages of autophagy. We have shown that ROC-325 does not affect the formation of autophagosomes but rather accumulates in and deacidifies the lysosome. In vitro treatment with ROC-325 shows stabilization of LC3-II and p62, indicators that are consistent with autophagy inhibition. In addition, treatment with ROC-325 results in near-complete loss of acridine orange fluorescence, a strong marker for lysosome membrane permeability and lysosome deacidification. ROC-325 reduced cell viability in RCC cell lines at much lower concentrations than HCQ, with half maximal inhibitory concentration (IC50) values of 2–10 μM vs. 50–100 μM, respectively.

In vivo experiments also demonstrated the improved anticancer activity of ROC-325 over HCQ. Mice treated with orally-administered ROC-325 displayed significantly decreased 786-O RCC xenograft burden when compared to both control and HCQ-treated mice. Importantly, no significant toxicities were observed in mice treated with ROC-325. Tumors taken from each group were analyzed using immunohistochemistry (IHC). Tumors treated with ROC-325 showed elevated levels of LC3-II, p62, and cleaved caspase-3, thereby confirming in vitro findings. Further study of ROC-325 especially in combination with conventional and targeted therapy is warranted.

### 4.4. STF-62247

STF-62247 is an experimental agent that was first discovered over a decade ago. This particular compound was identified to have potent cytotoxic effects in VHL-deficient cancer cells, but very little efficacy in wild-type (WT) VHL cells [45]. Due to this selective anti-tumor activity, this compound is a potentially exciting therapeutic option for RCC. The exact mechanism by which STF-62247 acts is not fully understood. STF-62247 is believed to induce autophagy in cancer cells, as treatment produces large, cytoplasmic vacuoles in both WT-VHL and VHL-deficient cells. However, VHL-deficient cells contain much larger vacuoles and show significantly brighter acridine orange staining [45]. This indicates that VHL-deficient cells form large, highly acidic vesicles in response to STF-62247. Upon further investigation, it was shown that Golgi vesicle trafficking proteins played a pivotal role in sensitizing cells to the compound. However, the mechanistic links between VHL and autophagy were not fully elucidated.

Bouhamdani et al. recently confirmed these findings in RCC and also noted that while VHL-proficient cells also form large vacuoles, they are capable of resolving them within 48 hours of treatment [44]. Interestingly, in this study, STF-62247 was not shown to induce autophagy, but rather blocked the late stages of autophagy. No known upstream markers of increased autophagy were shown to be affected by the drug and inhibiting the vacuolar H^+^ ATPase pump with bafilomycin A1 (BAF) showed very little, if any, additive stabilization of LC3-II or p62 when combined with STF-62247. These results cast doubt on the idea that STF-62247 is inducing autophagy. This also suggests that STF-62247 is potentially obstructing a similar stage of autophagy as BAF, indicating that it is a late-stage autophagy inhibitor, much like HCQ, CQ and ROC-325. However, much of this work is still controversial as STF-62247 has been shown to induce an autophagy-dependent cell death response in multiple malignancy types regardless of VHL status [61,62,63]. More data is needed in order for STF-62247 to be effectively transitioned into the clinical setting.

### 4.5. Lys05, DQ661, and DC661

Lys05 is a lysosome-disrupting, water-soluble salt of the compound Lys01. Lys01 consists of a pair of aminoquinolines, the major motif of CQ, connected by a methylamine-containing spacer [64,65]. Much like the previously discussed compounds, Lys01 produces an accumulation of LC3-II at a much lower dosage than CQ or HCQ. LN229 cells containing a green fluorescent protein tagged LC3 protein (GFP-LC3) treated with Lys01 display localization of LC3 to autophagic vesicles with 10x greater potency than HCQ, and electron microscopy confirms the presence of large autophagic vesicles in cells treated with Lys01. The anticancer profile of Lys01 is greater than that of HCQ when tested across colon cancer, glioma, and melanoma cell lines, with IC50 values ranging from 4–8 μM compared to 15–42 μM with HCQ. In vivo studies exhibited moderate, single agent, antitumor activity against orthotopically injected 1205Lu melanoma xenografts. Using HPLC tandem mass spectrometry, Lys05 was shown to accumulate in tumors in vivo at a much greater concentration than HCQ.

In addition to Lys05, two second-generation compounds have been developed—DQ661 and DC661 [66,67]. Interestingly, all these compounds were recently reported to block the lysosomal enzyme palmitoyl-protein thioesterase 1 (PPT1), which plays a key role in palmitoylation-mediated intracellular protein trafficking. These preclinical results imply that Lys05, DQ661, and DC661 have the potential to combat many different types of tumors. However, investigation of these compounds for RCC therapy has not been tested. It is worth noting that each of these lysosome-disrupting compounds has a similar mechanism of action, but contain unique chemical motifs. These novel structures will most likely lead to toxicity differences when clinical testing is initiated. This, in turn, could prove to be fundamental to approval and widespread therapeutic use.

### 4.6. VPS34 Inhibitors

The compounds discussed thus far act on the most distal component of the autophagy pathway, the lysosome. The lysosome acts as the common end point to this cellular process. The upstream, initiating machineries of autophagy show a great amount of complexity and have proven quite difficult to target. Nonetheless, multiple research groups are developing molecules to inhibit these proteins.

VPS34 is a PI3K lipid kinase family member, responsible for the addition of a phosphate group at the 3 position of the inositol ring of phosphatidylinositol (PI) on cellular membranes. This lipid phosphorylation is a vital step in the initiation and elongation of autophagosome membranes. While many pan-PI3K inhibitors currently exist, a few compounds have been developed to specifically target VPS34, including SAR405 and SB02024. SAR405 is a small molecule that targets the ATP binding pocket of VPS34 with high affinity [68,69]. In vitro work with this compound shows a reduction in autophagosome formation in GFP-LC3 tagged HeLa cells. In addition, concomitant treatment with everolimus in RCC cell lines eliminates the enhanced autophagic flux seen with mTOR inhibition. SAR405 also significantly enhanced the anticancer activity of everolimus in these same RCC cells. These preliminary findings have opened the door for combining VPS34 inhibitors with current standard-of-care therapeutics in RCC. SB02024, another recently discovered VPS34 inhibitor, shows significant anticancer activity when combined with standard RTK inhibitors, sunitinib and erlotinib, in breast cancer cell lines [70]. While this particular compound has not been tested in RCC, these findings highlight the potential of SB02024 to be paired with standard of care RCC agents.

### 4.7. ULK1 Inhibitors

UNC-51-like kinase 1 (ULK1) is a proximal serine/threonine kinase that is responsible for autophagy initiation. ULK1 acts as one of the key signaling regulators linking mTORC1, 5’ adenosine monophosphate-activated protein kinase (AMPK), starvation, and autophagy. Due to its vital role in integrating cellular stress signals, successful inhibition of ULK1 is an attractive therapeutic strategy. SBI-0206965 was developed in 2015 as a selective ULK1 inhibitor [71]. This compound displayed potent inhibition of ULK1 activity with an in vitro IC50 of 108 nM. Treatment of A549 human lung cancer cells in vitro with a combination of SBI-0206965 and the mTOR inhibitor AZD8055 led to a significantly enhanced apoptotic response. This result further supports the rationale of inhibiting autophagy to augment the activity of other target-specific molecules. More recently, two closely related compounds, ULK-100 and ULK-101, have also been developed as more potent inhibitors of ULK1 [72]. Martin et al. demonstrated that these two small molecules show increased antitumor activity when combined with nutrient starvation in non-small-cell lung cancer cell lines. All three of these novel compounds have the potential for therapeutic impact in RCC. However, their specific efficacy in RCC models has not yet been evaluated.

### 4.8. ATG4B Inhibitors

**Figure 2:**
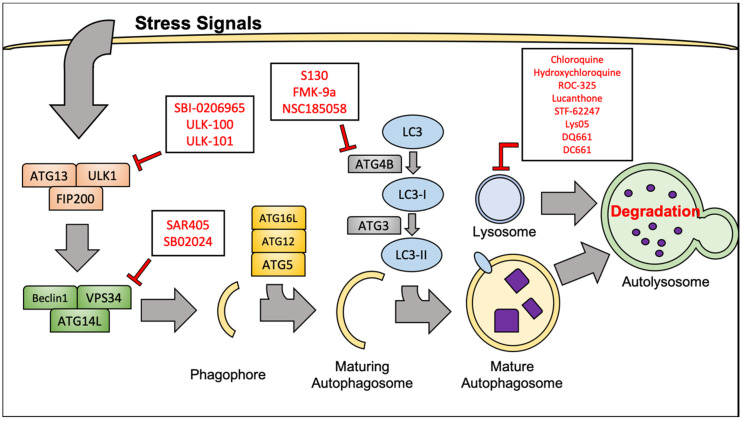
Selected agents that target autophagy at different points in the pathway. Hydroxychloroquine, chloroquine, and ROC-325 are amongst the compounds that target the lysosome. Compounds such as SBI-0206965 and SAR405 are being developed to inhibit autophagy factors near the proximal end of the cascade.

ATG4B is a cysteine protease that activates LC3 for lipidation and recent studies suggest that it may be another promising target to inhibit autophagy upstream of the lysosome. Consistent with this idea, several ATG4B inhibitors have been developed including FMK-9a, NSC185058, and S130 [73,74,75,76]. NSC185058 and S130 have demonstrated significant in vivo activity against osteosarcoma and colon tumors, respectively [74,75]. Additionally, NSC185058 treatment decreased glioma xenograft growth in mice and augmented the antitumor efficacy of radiation therapy [76]. Collectively, these studies demonstrate that inhibiting ATG4B may be a suitable anti-autophagy target for the treatment of various cancers. However, these compounds remain in the earliest stages of preclinical development (Figure 2).

## 5. Immune Checkpoint Inhibitor Therapy and Autophagy

The clinical efficacy of novel immune checkpoint inhibitors has been variable. Through further research and testing, cancer types are now generally thought of as “immune hot” and “immune cold”. Tumors that have significant infiltration of immune cells are considered “hot” and are generally responsive to immune checkpoint inhibitor therapy. The greatest immune checkpoint inhibitor therapy success to date has been the treatment of metastatic melanoma with a monoclonal antibody-targeting CTLA-4, Ipilimumab [77]. The FDA has now approved six different immune checkpoint inhibitors in a variety of cancer types. This number only stands to grow with over 200 active clinical trials involving immune checkpoint inhibitor therapies.

RCC is characterized as being responsive to immune checkpoint inhibitor therapy. As previously mentioned, cytokine therapy, a precursor to modern day immunotherapy, was long used as the primary treatment for advanced RCC. Nivolumab, a monoclonal antibody-targeting PD-1, was approved for use in RCC in 2015 after demonstrating improved progression-free survival (4.6 months vs. 4.4 months) over everolimus in a phase III clinical trial [26]. While more traditional small molecule therapy options, such as kinase inhibitors will remain a mainstay in RCC treatment, immunomodulatory therapies are becoming increasingly common as frontline and adjuvant therapies [78,79].

The connection between autophagy and immune cell activation is not well established, particularly in RCC. However, preliminary findings in different cancer models have provided conflicting findings. Early work has suggested that autophagy inhibition can interfere with hematopoiesis and systemic immunity indicating that combination autophagy and immune checkpoint inhibitor therapy may not be beneficial [80,81,82]. However, recent studies demonstrate that autophagy inhibition does not block T-cell activity [83,84,85,86]. Treatment of subcutaneous B16 melanoma xenografts with CQ in immunocompetent mice has provided evidence that autophagy inhibition promotes macrophage phenotype switching from an alternatively activated (M2) to a classically activated (M1) state [87]. This switch in macrophage phenotype gave rise to an increase in CD3+/CD8+ tumor-infiltrating lymphocytes as well as increased interferon gamma (IFNγ) expression, a key marker of cytotoxic T-lymphocyte (CTL) activation. Importantly, the antitumor effects of CQ in vivo were completely reversed in T-cell deficient mice, confirming that the activity of CQ was indeed a product of immunomodulation in this particular model. Recent work has also shown that HCQ, when delivered to E.G7-OVA murine lymphoma xenografts via nanoparticle vaccination, is capable of enhancing tumor cell major histocompatibility complex (MHC)-I antigen presentation, a key event in CTL activation [88]. A significant increase in the production of IFNγ was also observed in this model. Both of these recent studies highlight a potential relationship between autophagy inhibition and responsiveness to immune checkpoint inhibitor therapy, but additional studies are needed to better understand this interaction. Furthermore, the potential benefit of dual immune checkpoint and autophagy inhibitor therapy to RCC remains to be determined.

## 6. Conclusions

Two decades ago, patients diagnosed with advanced, metastatic, or surgically unresectable RCC had very few approved therapeutic options. However, significant research efforts have resulted in the development of numerous targeted agents and immune-related therapies for the treatment of RCC. Despite this success, patients that are refractory to these treatments or develop drug resistance continues to be a major clinical issue. Autophagy has now been established as a key mechanism by which cancer cells are capable of surviving periods of therapy-induced stress leading to drug resistance. This provides the rationale for the development and testing of autophagy-modulating compounds to use in conjunction with the ever-expanding list of approved RCC treatments. While HCQ has demonstrated some promising activity in combination with standard agents in clinical trials, its effectiveness appears to be limited by a variety of factors. Considering this, there is a need for new and more potent autophagy inhibitors that can be tested in clinical trials. Additional information is also required to determine the differences between upstream and lysosomal autophagy targeting in regards to therapeutic efficacy. The development and classification of compounds targeting autophagy is an ongoing process, but one can hope that a breakthrough is on the horizon.

## Figures and Tables

**Table 1:** FDA-approved treatments for RCC.

Category	Therapeutic Name	Target(s)	Comparator	PFS (in Months) vs. Comparator
Small Molecule Kinase Inhibitors	Axitinib [14]	VEGF, PDGF	Sorafenib	6–7 vs. 4–7
	Cabozantinib [15]	VEGFR-1,2,3, MET, FLT3, TIE-2, AXL, TRKB	Everolimus	7.4–9.1 vs. 3.7–5.1
	Erlotinib [16]	EGFR	Bevacizumab	9.9 vs. 8.5
	Lenvatinib [17]	VEGFR2	Everolimus	7.4 vs. 5.5
	Pazopanib [18]	VEGFR-1,2,3, PDGFR, c-kit	Placebo	9.2 vs. 4.2
	Sorafenib [19]	RAF, VEGFR, PDGFR	Placebo	5.5 vs. 2.8
	Sunitinib [20]	VEGFR2, PDGFRb, c-kit, FLT3	Interferon-alpha	11 vs. 5
mTOR Inhibitors	Everolimus [21]	FKBP-12	Placebo	4 vs. 1.9
	Temsirolimus [22]	mTOR	Interferon-alpha	5.5 vs. 3.1
Monoclonal Antibodies	Avelumab [23]	PD-L1	Sunitinib	13.8 vs. 8.4
	Bevacizumab [24]	VEGF	Interferon-alpha	10.2 vs. 5.4
	Ipilimumab [25]	CTLA4	Sunitinib	11.6 vs. 8.4
	Nivolumab [26]	PD-1	Everolimus	4.6 vs. 4.4
	Pembrolizumab [27]	PD-1	Sunitinib	15.1 vs. 11.1
Cytokine Therapy	Interferon alfa-2a [28]	Immunostimulatory	N/A	10% Response Rate
	Interleukin-2 [29]	Immunostimulatory	N/A	14% Response Rate

Abbreviations: VEGF—vascular endothelial growth factor; PDGF—platelet-derived growth factor; VEGFR—vascular endothelial growth factor receptor; MET—tyrosine-protein kinase Met; FLT3—fms like tyrosine kinase 3; TIE-2—angiopoietin-1 receptor; AXL—AXL receptor tyrosine kinase; TRKB—tropomyosin receptor kinase B; EGFR—epidermal growth factor receptor; RAF—rapidly accelerated fibrosarcoma; FKBP-12—FK506 binding protein 12; mTOR—mammalian target of rapamycin; PD-L1—programmed death ligand 1; CTLA4—cytotoxic t-lymphocyte associated protein 4; PD-1—programmed cell death protein 1; PFS—progression free survival.

**Table 2:** Clinical trials with hydroxychloroquine (HCQ) in patients with RCC.

Clinical Trial Identifier	Autophagy-Modulating Compound	Interventions	Phase	Neoplasm	DLTs	Response Rate
NCT01510119 [53]	HCQ	Everolimus	I/II	Previously Treated RCC	None in Phase I; Grades 3–4 AE’s <10%	SD or PR: 67%; Median PFS 6.3 Months
NCT01144169	HCQ	Surgery	I	Primary RCC	N/A	N/A
NCT01480154	HCQ	MK2206	I	Advanced Solid Tumors	N/A	N/A
NCT01550367	HCQ	IL-2	I/II	Metastatic RCC	Grades 3–5 AE’s 96.6%	SD/PR/CR: 69%; Median PFS 5.5 Months
NCT01023737 [54]	HCQ	Vorinostat	I	Advanced Solid Tumors	Grades 3–4 AE’s 18.5%	RCC Patient: PR for >50 cycles

**Table 3:** Selected agents that inhibit autophagy.

Inhibitor	Autophagy Target	Cancer Type	References
Hydroxychloroquine	Lysosome	RCC, etc.	[21,53,54,55]
Chloroquine	Lysosome	RCC, etc.	[51,52]
ROC-325	Lysosome	RCC, AML	[57,58,59,60]
Lucanthone	Lysosome	Breast	[41,56]
STF-62247	Lysosome	RCC, Glioblastoma, T-cell Leukemia	[45,61,62,63]
Lys05, DQ661, DC661	Lysosome, PPT1	Melanoma, Colon, Glioma	[64,65,66,67]
SAR405, SB02024	VPS34	RCC, Cervical	[68,69,70]
SBI-0206965, ULK-100, ULK-101	ULK1	Lung	[71,72]
S130, FMK-9a, NSC185058	ATG4B	Cervical, Colon, Osteosarcoma, GBM	[73,74,75,76]

Abbreviations: PPT1—palmitoyl-protein thioesterase 1; VPS34—vacuolar protein sorting 34; ULK1—unc51-like-kinase 1; ATG4B—autophagy related 4B; AML—acute myeloid leukemia; RCC—renal cell carcinoma; GBM—glioblastoma.

## Abbreviations

The following abbreviations are used in this manuscript:

RCC	renal cell carcinoma
VHL	Von-Hippel Lindau tumor suppressor
VEGF	vascular endothelial growth factor
PDGF	platelet-derived growth factor
ATG	autophagy-related gene
PFS	progression-free survival
PR	partial response
DLT	dose-limiting toxicity
SD	stable disease
CR	complete response
HDAC	histone deacetylase
AE	adverse event
HCQ	hydroxychloroquine
CQ	chloroquine
IHC	immunohistochemistry
ULK	UNC-51 like kinase
CTLA-4	cytotoxic t-lymphocyte associated protein 4
PD-L1	Programmed cell death ligand 1
